# Relationship Between the Lactate-to-Albumin Ratio and Acute Kidney Injury in Patients with Pulmonary Embolism: A Retrospective Cohort Study

**DOI:** 10.3390/medicina62030474

**Published:** 2026-03-02

**Authors:** Dogan Ilis, Ayca Arslan, Inanc Artac, Muammer Karakayali, Omer Kertmen, Hatice Taskan, Yuksel Erata, Ezgi Guzel, Yavuz Karabag, Ibrahim Rencuzogullari

**Affiliations:** 1 Department of Cardiology, Kafkas University Faculty of Medicine, 36000 Kars, Turkey; hivda.arslan@hotmail.com (A.A.); inancartac@hotmail.com (I.A.); muammer-28@hotmail.com (M.K.); yukselerata61@gmail.com (Y.E.); ezggzl@hotmail.com (E.G.); yavuz_karabag@hotmail.com (Y.K.); rencuzog@gmail.com (I.R.); 2 Department of Cardiology, Faculty of Medicine, Amasya University, 05100 Amasya, Turkey; omerkertmen@gmail.com; 3 Department of Cardiology, Haseki Training and Research Hospital, University of Health Sciences, 34265 Istanbul, Turkey; haticetaskan@gmail.com

**Keywords:** pulmonary embolism, lactate-to-albumin ratio, acute kidney injury

## Abstract

*Background and Objectives*: Pulmonary embolism (PE), the third most prevalent cause of cardiovascular death, is often regarded as a potentially fatal condition. Renal function has been shown to affect the short- and long-term prognosis of acute PE in several large registries. Therefore, the purpose of this study is to investigate the relationship between acute kidney injury (AKI) and the lactate-to-albumin ratio (LAR) in patients hospitalized for PE. *Materials and Methods*: 264 PE patients were included in this retrospective analysis. Based on the presence or absence of AKI, the study population was split into two groups. *Results*: Of the 264 patients included in our study, 161 (61%) were female. The median age was 67 ± 16 years. The sample was divided into two groups based on whether AKI developed (No AKI group, *n* = 176; AKI group, *n* = 88). A multivariate logistic regression analysis revealed that sPESI score, receiving fibrinolytic therapy (tPA), and LAR (OR: 6.334, 95% CI: 3.070–13.069; *p* < 0.001) were independently associated with AKI in patients with PE. In particular, an LAR > 0.55 predicted AKI in patients with PE, with a sensitivity of 75% and a specificity of 67% (AUC = 0.749, 95% CI = 0.692–0.800, *p* < 0.001). *Conclusions*: Our study demonstrates that the LAR independently predicts AKI in patients with PE. This is the first study that precisely examines this association in PE patients, as far as we are aware.

## 1. Introduction

Pulmonary embolism (PE), the third most prevalent cause of cardiovascular death, is often regarded as a potentially fatal condition [1]. The mortality rate for high-risk PE patients with hemodynamic instability is still as high as 14%, despite a decline in the overall mortality rate [2,3,4,5]. Rapid and accurate risk assessment is essential, since PE is extremely life-threatening. Renal function has been shown to impact the short- and long-term prognosis of acute PE in a number of significant registries. One of the widely recognized markers of a higher death rate in a number of cardiovascular conditions is renal impairment. Renal failure was an independent predictor of mortality in the International Cooperative Pulmonary Embolism Registry study [6].

The main cause of death in acute PE is systemic hypotension and hypoperfusion, which are brought on by failure in the right heart, which is typically accompanied by a reduction in left ventricular filling and cardiac output [1]. Cellular hypoxia is worsened by hypoxemia brought on by a ventilation/perfusion scan mismatch. Anaerobic metabolism produces lactate, which has been shown to indicate tissue hypoperfusion and the severity of cellular hypoxia. Additionally, it can forecast the mortality rate and organ failure in critically ill patients [7,8]. However, abnormal lactate levels can also be caused by metformin consumption, improper proteolytic metabolism, or liver damage [9,10].

The inhibition of platelet aggregation, preservation of endothelial integrity, and anti-inflammatory action are among the physiological effects of albumin [11,12]. Previous research has shown that patients with low albumin levels have a higher risk of restenosis following coronary stent implantation [13]. Thus, hypoalbuminemia’s prothrombotic condition may be a factor in higher mortality. Additionally, serum albumin has a predictive significance; hypoalbuminemia, commonly defined as less than 35 g/L, is prevalent in critically ill patients and is linked to worse outcomes [14,15]. Recently, the lactate-to-albumin ratio (LAR) has become a useful prognostic index, showing predictive value in conditions like severe sepsis, septic shock, pulmonary embolism, and cardiac arrest [16,17,18,19].

Acute kidney injury (AKI) is common in patients with pulmonary embolism [20,21,22], and AKI can be diagnosed in up to one-third of patients [23]. The AKI can occur in various clinical scenarios and may increase all-cause mortality [23,24,25]. This is associated with increased mortality in the short to medium term [23,26]. Predicting this outcome beforehand can provide an advantage in the event of anticipated poor clinical outcomes. Although LAR has been evaluated in various patient populations for its ability to predict adverse outcomes, there has not been enough research performed on the relationship between the LAR and AKI outcomes in PE patients. Therefore, the purpose of this study is to investigate the relation between AKI and the LAR in patients hospitalized for pulmonary embolism.

## 2. Materials and Methods

### 2.1. Population and Sample

**Figure 1:**
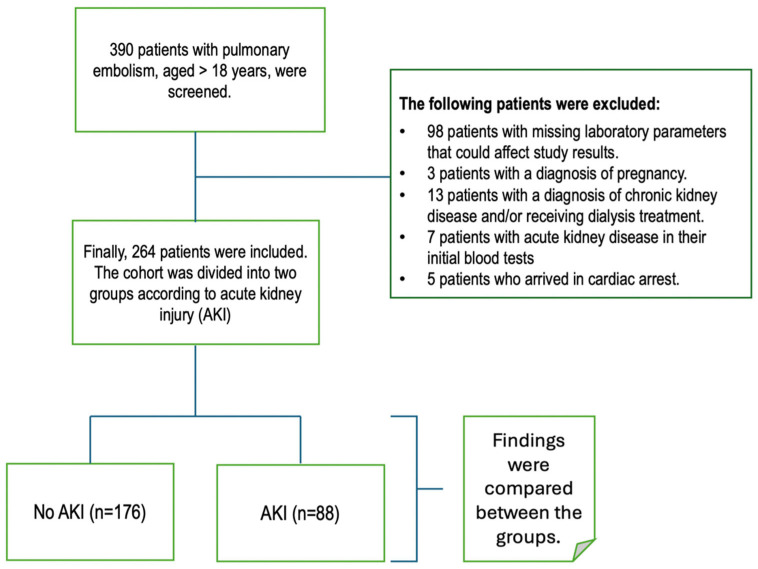
Flow chart of the study sample selection.

In this study, patients admitted to our institution due to PTE (confirmed by CT pulmonary angiography (CTPA)) between September 2020 and September 2024 were retrospectively reviewed. A total of 390 patients aged 18 or older were screened. After excluding 98 patients with missing laboratory parameters that could affect study results, 3 patients with a diagnosis of pregnancy, 13 patients with a diagnosis of chronic kidney disease and/or receiving dialysis treatment, 7 patients with acute kidney disease in their initial blood tests, and 5 patients who arrived with cardiac arrest, 264 adult patients were included in the study (Figure 1). The local ethics committee approved the study protocol before the study began. The investigation was carried out in compliance with the ethical criteria established by the authorized ethics committee on human testing, as well as the ethical principles outlined in the Declaration of Helsinki.

### 2.2. Data Extraction

Retrospective data from the patients’ charts were used to compile their medical histories, including comorbidities and echocardiographic information. Blood samples were obtained from all patients upon hospital admission or hospitalization for complete blood count (CBC) and measurement of biochemical markers. For CBC tests and biochemical marker detection, an automated hematological and biochemical analyzer (Roche Diagnostics Cobas 8000 c502, Indianapolis, IN, USA) was utilized. We chose two main indicators at the start of the admission process, namely blood lactate concentration and serum albumin level. The initial results were obtained within 24 h of hospital admission, with blood lactate measured from arterial blood and serum albumin from venous blood.

### 2.3. Definition of Acute Kidney Injury

The hospital admission date was taken into consideration, and according to information obtained from archive searches, acute kidney disease (AKD) was defined as a GFR < 60 mL/min per 1.73 m^2^ detected in kidney function tests within the last 3 months. (AKD, GFR < 60 mL/min per 1.73 m^2^ for <3 months.)

According to information obtained from archive searches, chronic kidney disease (CKD) was defined as a GFR < 60 mL/min per 1.73 m^2^ detected in kidney function tests for more than 3 months. (CKD, GFR < 60 mL/min per 1.7m^2^ for ≥3 months.)

Patients for whom this information could not be obtained were excluded.

Acute kidney injury (AKI) was assessed using the “Kidney Disease: Improving Global Outcomes” definition (KDIGO) in hospitalized patients diagnosed with pulmonary embolism [27].

AKI is defined by KDIGO as the existence of any of the following:

1. A rise in serum creatinine within 48 h of at least 0.3 mg/dL (26.5 μmol/L).

2. A rise in serum creatinine to at least 1.5 times the previous seven days’ baseline.

3. Less than 0.5 mL/kg/h of urine for a minimum of six hours.

For the ≥1.5× within 7 days criterion, the most recent stable serum creatinine value obtained within 7 days prior to admission was used as the baseline; if unavailable, admission creatinine was used as the reference for subsequent in-hospital comparisons. Serum creatinine levels were measured at admission and monitored at 24-h intervals during hospitalization. Due to the retrospective design and incomplete urine output data, AKI diagnosis in the present study was based exclusively on serum creatinine criteria.

### 2.4. sPESI Calculation

A history of cancer and chronic cardiopulmonary disease, age > 80 years, heart rate ≥ 110 bpm, systolic blood pressure < 100 mm Hg, and arterial oxygen saturation < 90% at diagnosis were among the factors used to compute the sPESI score [28]. Patients with missing data for any of the variables required to calculate the sPESI score were excluded from the study. Heart failure and chronic lung disease are examples of chronic cardiopulmonary diseases. A left ventricular ejection fraction of less than 40%, symptoms consistent with heart failure (New York Heart Association class ≥ 2), or a history of hospitalization for the condition were used to diagnose heart failure. Asthma, COPD, and restrictive lung disorders are examples of persistent lung conditions that are classified as chronic lung diseases.

### 2.5. Statistical Analysis

Statistical analyses were conducted using SPSS 22.0 (Statistical Product and Service Solutions for Windows, Version 22.0, IBM Corp., Armonk, NY, USA, 2013). For continuous variables that followed a normal distribution, descriptive statistics are expressed as means ± SDs; continuous variables that did not are expressed as medians with 0.25 and 0.75 quantiles; and categorical variables are expressed as percentage values. Fisher’s exact test or the chi-squared test was used for categorical variables, and the *t*-test or Mann–Whitney U test was used to evaluate continuous variables between groups.

Univariate binary logistic regression analyses were conducted for all clinically relevant variables potentially predicting AKI. Multivariate binary logistic regression analysis, using stepwise backward conditional elimination, was performed on the variables found to be significant in univariate analyses (*p* < 0.05) to determine the independent predictors of AKI. The LAR was analyzed as a continuous variable using the multivariate logistic proportional odds model. Multicollinearity between the LAR and its components (lactate and albumin levels) was assessed using the eigenvalue and condition index. Linearity was tested through interaction with the logarithmic transformation of each parameter. Receiver operating characteristic (ROC) analysis was performed to determine the optimal LAR cutoff for predicting AKI. A multivariate binary logistic regression model was constructed to assess the independent and combined predictive value of the two parameters. Model-derived predicted probabilities served as the test variable for receiver operating characteristic (ROC) curve analysis. Discriminatory performance was quantified using the area under the curve (AUC) with 95% confidence intervals.

## 3. Results

Of the 264 patients included in our study, 161 (61%) were female. The median age was 67 ± 16 years. The overall incidence of post-pulmonary embolism AKI in our study population was 33.3%. Based on whether or not AKI developed, the sample was divided into two groups (AKI group, *n* = 88; no AKI group, *n* = 176).

Patients in the AKI group were older (72 ± 12 vs. 64 ± 17 years, *p* = 0.001) and had a significantly higher mortality rate [18 (20.5%) vs. 3 (1.7%), *p* < 0.001]. They also had a higher prevalence of hypertension [50 (56.8%) vs. 76 (43.2%), *p* = 0.037] and chronic CPD [32 (36.4%) vs. 40 (22.7%), *p* = 0.019]. Additionally, these patients showed higher elevated lactate levels [2.4 (1.8–3.5) vs. 1.7 (1.2–2.6), *p* < 0.001]. From the perspective of medication use, it was found that these patients had a higher rate of beta-blocker use [28 (31.8) vs. 36 (20.5), *p* = 0.043]. Again, a higher rate of receiving fibrinolytic therapy (tPA) was observed among these patients (17 (19.3) vs. 13 (7.4), *p* = 0.004). These patients also had higher sPESI scores [3 (2–4) vs. 2 (2–3), *p* < 0.001] and a higher LAR [0.68 (0.53–1.25) vs. 0.42 (0.29–0.66), *p* < 0.001]. However, patients with AKI had significantly lower albumin levels (3.51 ± 0.52 vs. 3.93 ± 0.48, *p* < 0.001) and mean LVEF (55 ± 9 vs. 58 ± 7, *p* < 0.001) (Table 1).

A univariate logistic regression analysis indicated associations between AKI and factors such as age, hypertension, LVEF, beta-blocker use, tPA, LAR, and sPESI score. Subsequently, factors identified as significant in the univariate analysis were included in the multivariate model. A multivariate logistic regression analysis revealed that the sPESI score, tPA, and LAR (OR: 6.319, 95% CI: 3.009–13.268; *p* < 0.001) were independently associated with AKI in patients with PE (Table 2).

**Figure 2:**
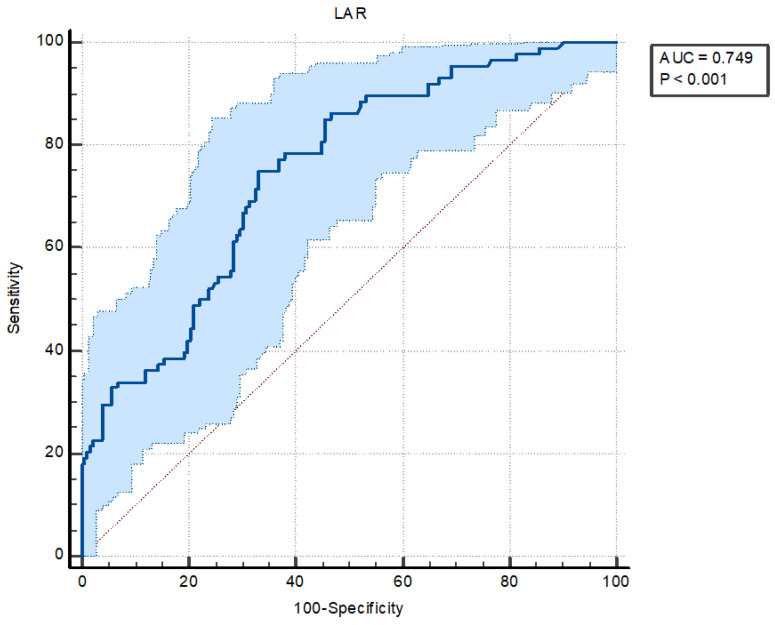
The receiver operating characteristic (ROC) curve analysis of LAR in predicting acute kidney injury in patients with pulmonary embolism. (LAR: AUC: 0.749, Cut-off > 0.55 Sensitivity: 75% Specificity: 67%) Abbreviations: LAR, lactate to albumin ratio.

**Figure 3:**
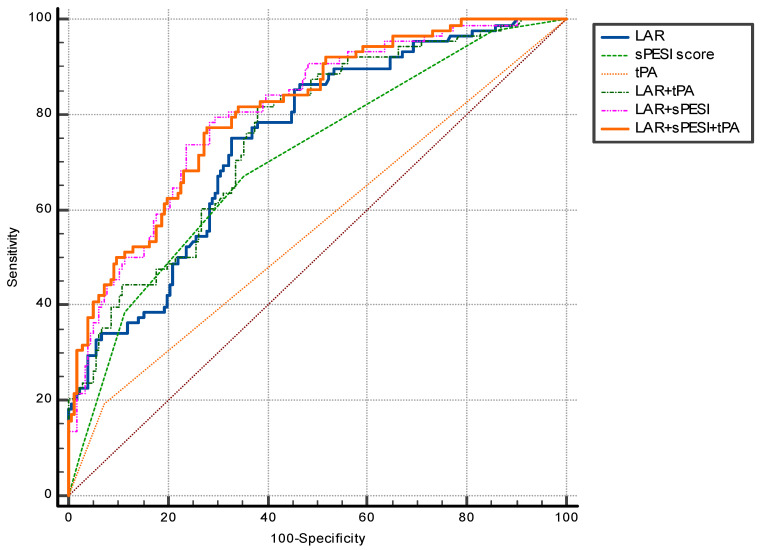
The receiver operating characteristic (ROC) curve analysis of sPESI, tPA, LAR + sPESI, LAR + tPA, and LAR + sPESI + tPA in predicting acute kidney injury in patients with pulmonary embolism. Abbreviations: LAR, lactate to albumin ratio; sPESI, simplified pulmonary embolism severity index; tPA, tissue plasminogen activator.

The ROC curve analysis identified optimal cutoff values for the LAR, which were independent predictors of AKI in the multivariate analysis. In particular, an LAR > 0.55 predicted AKI in patients with PE, with a sensitivity of 75% and a specificity of 67% (AUC = 0.749, 95% CI = 0.692–0.800, *p* < 0.001) (Figure 2). The AUC values for sPESI and tPA were 706 and 0.560, respectively (AUC = 706, 95% CI = 0.640–0.773, *p* < 0.001; AUC = 0.560, 95% CI = 0.484–0.635, *p* = 0.114 respectively). The ROC curve analysis also identified the Combining the LAR + sPESI, LAR + tPA, and LAR + sPESI + tPA. Combining the LAR + sPESI, LAR + tPA, and LAR + sPESI + tPA also yielded a significantly higher accuracy in predicting AKI (AUC = 0.805, 95% CI = 0.751–0.860, *p* < 0.001; AUC = 0.759, 95% CI = 0.700–0.819, *p* < 0.001; AUC = 0.808, 95% CI = 0.754–0.862, *p* < 0.001 respectively) (Figure 3).

## 4. Discussion

Our study demonstrates that the LAR independently predicts AKI in patients with PE. To the best of our knowledge, this is the first study to explore this relationship specifically in patients with PE.

PE is a clot that forms in the pulmonary arteries and their branches, typically due to the migration of a deep vein thrombus. It is the third most common cause of cardiovascular death globally, behind stroke and coronary artery disease [29,30]. Although the rates of AKI reported in PE patients vary in studies, these rates were found to be between 35% and 65% [23,31,32]. In our research, the AKI rate was 33.3%. These rates may have varied depending on the different clinical settings of the patients included in the study and the selection of patients who were only admitted to the intensive care unit—for example, in our study, we included patients who were admitted to the inpatient clinic or the intensive care unit in our study. AKI in PE patients has a complex pathogenesis that remains unclear. Lower renal perfusion pressure in high-risk PE patients can lead to renal hypoperfusion and increase the risk of AKI [33]. Right ventricular dysfunction in PE patients can increase central venous pressure and lead to renal venous hypertension [34]. Acute right ventricular dysfunction and tricuspid problems, including regurgitation, can result from sudden pressure overload in the pulmonary circulation brought on by PE. Central venous pressure increases as a result, which could cause renal congestion and subsequent damage [23,35]. Reduced preload caused by right ventricular failure decreases left ventricular output. These acute conditions result in hypoperfusion of the renal macrocirculation and/or microcirculation, thereby triggering injury. Cardiogenic shock and the ensuing hypoperfusion of the kidneys can also happen [35].

Lactate serves as a key indicator of tissue oxygenation, metabolism [36], and blood perfusion [37], while albumin reflects nutritional status [38] and inflammation [39]. As a negative acute-phase reactant, albumin levels correlate with inflammation severity, disease prognosis, and mortality. Low albumin is a known marker of adverse outcomes in cardiovascular conditions such as heart failure [40], myocardial infarction [41], stroke [42], and pulmonary embolism [32]. The LAR, which is the ratio of these two markers, captures their opposing regulatory effects, helping to overcome the limitations of relying on a single marker. Xu J et al. [16], in their analysis of the MIMIC-IV database, examined the relationship between LAR and 28-day mortality in patients with PE. They found that high LAR levels are directly associated with increased mortality and that this ratio serves as an independent prognostic indicator. Their study showed that levels above the 0.67 threshold they established for LAR carry an increased risk of death. In their study of 8661 sepsis patients, Wang J. et al. reported a positive correlation between early elevated LAR levels and sepsis-induced coagulopathy and short-term (28-day) and long-term (360-day) mortality. The median LAR value was 0.7 across all sepsis patients [43]. In a multicenter study, the predictor of mortality risk in patients with AKI treated in intensive care units (ICUs) was investigated using the LAR value. Patients were divided into four quartiles based on their LAR values (quartiles Q1 (LAR < 0.46, *n* = 1167), Q2 (0.46 ≤ LAR < 0.79, *n* = 1162), Q3 (0.79 ≤ LAR < 1.49, *n* = 1170), and Q4 (LAR ≥ 1.49, *n* = 1167)). The study revealed that high LAR levels were an independent risk factor for both in-hospital and ICU deaths [44]. Our study is the first to directly examine in-hospital acute renal failure in patients with pulmonary embolism using the LAR value obtained at hospital admission, and we established a threshold of 0.55. Our study showed a relationship between an elevated LAR and AKI, independent of mortality, in PE patients. The association of AKI with increased mortality and longer hospital stay in PE patients is well known. The LAR is simple, inexpensive, and easily accessible, and we believe that the clinical practical advantages it will provide in identifying at-risk patients are significant. The LAR, a readily available and quantifiable biomarker, shows promise as a tool for identifying patients at risk for AKI in patients with PE. Consequently, high LAR values (e.g., above 0.55 based on our data) indicate patients who may be at higher risk, supporting more vigilant monitoring and earlier intervention. Identifying patients with an LAR > 0.55 at admission may prompt closer renal function monitoring, optimizing hemodynamic status, the cautious use of nephrotoxic agents, and more judicious fluid and contrast management. Additionally, the LAR may complement existing clinical and laboratory parameters to guide early multidisciplinary decision-making and individualized patient management. We believe that the simplicity and widespread availability of the LAR enhance its potential utility in routine clinical settings.

The sPESI score was initially designed to predict 30-day mortality in patients with acute PE [28,45]. However, in our study, we evaluated patients with embolism for the development of AKI during hospitalization. Because sPESI is a validated score that provides a comprehensive clinical assessment, it was calculated for patients through archival research. In our study cohort, we showed that the LAR and sPESI score were independent predictors of AKI in patients with PE. We think that multicenter, prospective trials are necessary to clearly confirm the validity of the LAR.

Kidney health is significantly impacted by age. Age-related decreases in the glomerular filtration rate, nephron number, and tubular epithelial cells can increase susceptibility to AKI [46]. Age is also a significant risk factor for AKI [47]. However, age is associated with 30-day mortality in PE and is incorporated into prognostic scoring systems [45]. In older individuals, renal parenchymal regeneration significantly declines, and fibrosis develops as a result of the overproduction of the extracellular matrix in the kidney interstitium [48]. Declined renal function with age is multidimensional and undoubtedly increases susceptibility to AKI [49]. Our results are consistent with existing research, indicating that the AKI group had a notably higher average age.

HT is a risk factor for AKI. HT increases the risk of developing kidney disease through sodium/water retention, the renin–angiotensin system, sympathetic nervous system activation, and/or endothelial dysfunction [50]. HT is associated with an increased risk of AKI in hospital admissions, independent of blood pressure [51,52,53]. Adverse changes caused by HT in the chronic renal process may be related to larger fluctuations in blood pressure and the inability of patients to more rapidly stabilize their blood pressure within an appropriate range. In line with the literature, the study population had a higher proportion of patients diagnosed with HT among those who developed AKI. Major thrombosis involving more than 50% of the pulmonary arteries, known as major pulmonary embolism (PE), is a difficult clinical problem. These patients are usually hemodynamically unstable and should be immediately diagnosed and treated [54]. In our study population, the proportion of patients receiving tPA treatment was higher in the AKI group. We believe that this is due to impaired renal perfusion caused by massive embolism or hemodynamic instability, which initially led to the indication for tPA treatment. Finally, when we consider the amount of contrast agent used and the differences between the groups, CTPA scans were performed according to a standard imaging protocol. In our study population, no statistically significant difference in the amount of contrast agent was found between the groups.

### Limitations

Despite our significant findings, the study had some limitations. First, our statistical power was constrained by the small sample size (264 patients). Furthermore, the generalizability of our findings from a single inpatient clinic and intensive care unit cohort to outpatients or milder cases of pulmonary embolism remains unclear. Second, we focused only on the relationship between the LAR and outcomes at a single time point after admission, ignoring the effect of the dynamic variation in the LAR on prognosis. Third, the retrospective nature of the study limits the possibility of repeating the analysis. Fourth, a limitation of this study is the absence of a comparison between included and excluded patients. Because lactate and albumin measurements were unavailable for all screened individuals, exclusions may have been influenced by clinical severity or institutional testing practices, introducing potential selection bias. Therefore, the findings should be interpreted with caution and confirmed in prospective studies with systematic biomarker assessment. Finally, we did not compare the diagnostic value of LAR in patients with pulmonary embolism with other markers of acute renal failure. To confirm the prognostic utility of the LAR for predicting AKI in pulmonary embolism, prospective research including larger and more varied populations is required. Additionally, independent, multicenter external validation studies are needed to confirm the generalizability of the identified 0.55 LAR cutoff value.

## 5. Conclusions

In summary, we observed a significant association between admission LAR levels and the development of AKI in patients with PE. Although LAR remained associated with AKI after adjustment for selected clinical and hemodynamic variables, it may partially reflect overall disease severity and hemodynamic compromise. Therefore, LAR should be interpreted as a potential risk marker rather than a definitive independent predictor. Larger prospective studies with more comprehensive control of severity-related factors are required to clarify its independent prognostic value and its potential role in clinical practice.

## Figures and Tables

**Table 1:** The baseline demographic, laboratory, and clinic characteristics of the study population according to the acute kidney injury.

Variables		No AKI (*n* = 176, 66,7%)	AKI (*n* = 88, 33.3%)	Study Population (*n* = 264)	*p*
Age, years		64 ± 17	72 ± 12	67 ± 16	**0.001**
Female, *n* (%)		111 (63)	50 (57)	161 (61)	0.327
Coronary artery disease, *n* (%)		27 (15.3)	19 (21.6)	46 (17.4)	0.208
Hypertension		76 (43.2)	50 (56.8)	126 (47.7)	**0.037**
Diabetes mellitus, *n* (%)		31 (17.6)	19 (21.6)	50 (18.9)	0.438
CVA/TIA, *n* (%)		8 (4.5)	3 (3.4)	11 (4.2)	0.664
Hyperlipidemia, *n* (%)		12 (6.8)	8 (9.1)	20 (7.6)	0.511
Current smoker, *n* (%)		38 (21.6)	19 (21.6)	57 (21.6)	1.000
In hospital mortality, *n*;(%)		3 (1.7)	18 (20.5)	21 (8.0)	**<0.001**
Hospitalization duration, days (IQR)		8 (7–10)	9 (7–12)	8 (7–11)	**0.023**
Beta Blockers, *n* (%)		36 (20.5)	28 (31.8)	64 (24.0)	**0.043**
ACEI, *n* (%)		36 (20.5)	18 (20.5)	54 (20.5)	1.000
ARB, *n* (%)		29 (16.5)	20 (22.7)	49 (18.6)	0.219
Statin, *n* (%)		26 (14.8)	13 (14.8)	39 (14.8)	1.000
Heart rate, bpm (SD)		98 ± 16	99 ± 16	98 ± 16	0.856
Systolic BP, mm/Hg (SD)		114 ± 20	112 ± 21	113 ± 121	0.087
Diastolic BP, mm/Hg (SD)		68 ± 10	65 ± 9	67 ± 9	0.810
Saturation, % (SD)		90 ± 5	88 ± 5	89 ± 5	0.074
WBC count, 10^3^/μL (SD)		11 ± 4	12 ± 5	12 ± 4	0.055
Hemoglobin, g/dL (SD)		13 ± 2	12 ± 2	13 ± 2	0.161
Glucose, mg/dL (SD)		135 ± 44	139 ± 41	137 ± 43	0.274
Creatinine, mg/dL (SD)		1.11 ± 0.27	1.12 ± 0.21	1.12 ± 0.25	0.954
BUN, mg/dL (IQR)		31 (22–44)	38 (18–53)	33 (21–48)	0.091
Troponin I, (ng/mL) (IQR)		40 (10–210)	70 (20–285)	50 (10–225)	0.072
ALT, U/L (IQR)		19 (14–35)	25 (14–88)	21 (14–41)	0.055
AST, U/L (IQR)		23 (18–37)	24 (18–38)	23 (18–37)	0.924
C-Reactive Protein, mg/dL (IQR)		45 (24–202)	58 (24–146)	45 (24–159)	0.578
D-dimer (mcg/mL) (IQR)		2.1 (1.02–3.7)	2.7 (1.2–4.4)	2.1 (1.1–3.9)	0.071
LAR (IQR)		0.42 (0.29–0.66)	0.68 (0.53–1.25)	0.51 (0.32–0.74)	**<0.001**
Lactate, (mmol/L) (IQR)		1.7 (1.2–2.6)	2.4 (1.8–3.5)	2.0 (1.3–2.8)	**<0.001**
Albumin, g/dL (SD)		3.93 ± 0.48	3.51 ± 0.52	3.79 ± 0.53	**<0.001**
Contrast volume, mL (SD)		89 ± 7	88 ± 5	89 ± 7	0.423
sPESI score (IQR)		2 (2–3)	3 (2–4)	2 (2–3)	**<0.001**
sPESI parameters	Age > 80 years, *n* (%)	28 (15.9)	35 (39.8)	63 (23.9)	**<0.001**
	Cancer, *n* (%)	25 (14.2)	16 (18.2)	41 (15.5)	0.401
	Chronic CPD, *n* (%)	40 (22.7)	32 (36.4)	72 (27.3)	**0.019**
	Pulse ≥ 110 bpm, *n* (%)	51 (29.0)	27 (30.7)	78 (29.5)	0.775
	SBP < 100 mmHg, *n* (%)	29 (16.5)	20 (22.7)	49 (18.6)	0.270
	SaO_2_ < 90%, *n* (%)	89 (50.6)	64 (61.4)	143 (54.2)	0.098
LVEF, % (SD)		58 ± 7	55 ± 9	57 ± 8	**0.001**
tPA, *n* (%)		13 (7.4)	17 (19.3)	30 (11.4)	**0.004**

Abbreviations: AKI, acute kidney injury; CVA: Cerebrovascular accident, TIA: Transient ischemic attack; ACEI, angiotensin-converting enzyme inhibitor; ARB, angiotensin receptor blocker, BP, blood pressure; WBC, white blood cell; BUN, Blood-urea-nitrogen; ALT, alanine transaminase; AST, aspartate aminotransferase; LAR, lactate to albumin ratio; sPESI, simplified pulmonary embolism severity index; CPD, cardiopulmonary disease; SBP, systolic blood pressure; LVEF, left ventricular ejection fraction; tPA, tissue plasminogen activator; SD, standard deviation; IQR, interquartile range.

**Table 2:** Univariate and Multivariate Analyses of Variables Predicting Acute Kidney Injury in Patients with Pulmonary Embolism.

	Univariate			Multivariate		
Variables	Odds Ratio	95% CI	*p* Value	Odds Ratio	95% CI	*p* Value
Age	1.033	1.014–1.053	**0.001**			
LAR	7.863	3.805–16.250	**<0.001**	6.319	3.009–13.268	**<0.001**
sPESI	2.183	1.652–2.885	**<0.001**	2.030	1.497–2.754	**<0.001**
LVEF	0.958	0.928–0.988	**0.007**			
Hypertension	1.731	1.033–2.902	**0.037**			
Beta-blocker	1.815	1.017–3.238	**0.044**			
tPA	3.002	1.384–6.510	**0.005**	3.022	1.222–7.469	**0.017**

Abbreviations: LAR, lactate to albumin ratio; sPESI, simplified pulmonary embolism severity index; LVEF, left ventricular ejection fraction; DBP, diastolic blood pressure; tPA, tissue plasminogen activator.

## Data Availability

Data are from Kafkas University Health Research and Application Center and are not available to the public as it may compromise the privacy of research participants. Further enquiries can be directed to the corresponding author (D.I.) upon reasonable request.
